# Leveraging implementation science to reduce inequities in Children’s mental health care: highlights from a multidisciplinary international colloquium

**DOI:** 10.1186/s12919-020-00184-2

**Published:** 2020-04-06

**Authors:** Nicole A. Stadnick, Gregory A. Aarons, Lucy Blake, Lauren I. Brookman-Frazee, Paul Dourgnon, Thomas Engell, Florence Jusot, Anna S. Lau, Constance Prieur, Ane-Marthe Solheim Skar, Miya L. Barnett

**Affiliations:** 1 University of California San Diego, La Jolla, CA 92093 USA; 2 Child and Adolescent Services Research Center, San Diego, CA 92123 USA; 3 University of California San Diego Dissemination and Implementation Science Center, La Jolla, CA 92093 USA; 4 Faculty of Health, Social Care & Medicine, Edge Hill University, Ormskirk, Lancashire UK; 5 Autism Discovery Institute, Rady Children’s Hospital, San Diego, USA; 6 Institut de Recherche et Documentation en Economie de la Santé, Paris, France; 7 Regional Centre for Child and Adolescent Mental Health, Eastern and Southern Norway, Oslo, Norway; 8 Université Paris-Dauphine, Paris, France; 9 University of California, Los Angeles, Los Angeles, CA 90095 USA; 10 Paris Descartes University School of Medicine, Paris, France; 11 The Norwegian Centre for Violence and Traumatic Stress Studies, Oslo, Norway; 12 University of California, Santa Barbara, Santa Barbara, CA 93106 USA

**Keywords:** Child, Mental health, Implementation science, Equity, International

## Abstract

**Background and purpose:**

Access to evidence-based mental health care for children is an international priority. However, there are significant challenges to advancing this public health priority in an efficient and equitable manner. The purpose of this international colloquium was to convene a multidisciplinary group of health researchers to build an agenda for addressing disparities in mental health care access and treatment for children and families through collaboration among scholars from the United States and Europe engaged in innovative implementation science and mental health services research.

**Key highlights:**

Guided by the Exploration, Preparation, Implementation, and Sustainment (EPIS) Framework, presentations related to inner, outer, and bridging context factors that impact the accessibility and quality of mental health evidence-based practices (EBPs) for children and families. Three common topics emerged from the presentations and discussions from colloquium participants, which included: 1) the impact of inner and outer context factors that limit accessibility to EBPs across countries, 2) strategies to adapt EBPs to improve their fit in different settings, 3) the potential for implementation science to address emerging clinical and public health concerns.

**Implications:**

The common topics discussed underscored that disparities in access to evidence-based mental health care are prevalent across countries. Opportunities for cross-country and cross-discipline learnings and collaborations can help drive solutions to address these inequities, which relate to the availability of a trained and culturally appropriate workforce, insurance reimbursement policies, and designing interventions and implementation strategies to support sustained use of evidence-based practices.

## Background

Early prevention and intervention for children at risk for or experiencing emerging symptoms of mental health disorders is critical to maximize treatment benefits and upstream effects on quality of life [1]. A meta-analysis found that worldwide 13.4% of children and adolescents have mental disorders [2]. Of those affected, the vast majority of children and adolescents (75% of youth in low- and middle-income countries) do not receive mental health treatment; when they do receive treatment, it is unlikely to be evidence-based [3, 4]. There are a growing number of evidence-based practices with demonstrated efficacy to address youth mental health disorders and problems [5].. Unfortunately, access to these EBPs and early intervention are often limited due to challenges implementing them in health and allied health settings, which impedes the potential public health impact of these interventions [6]. Underserved communities, including ethnic and racial minority communities, immigrants, and individuals residing in low- and middle income countries, are even less likely to have services available or be able to access high quality, evidence-based treatments [3, 4, 7]. Further complicating matters, there are emerging areas of clinical concern and public health attention that are not directly addressed by current EBPs such as youth screen time and family estrangement [8, 9]. Accessing and tailoring mental health care for youth and families from underserved backgrounds is even more challenging for children with clinically complex needs, such as children with autism spectrum disorder [10]. Therefore, it is critical to understand how to decrease disparities in mental health for vulnerable children and families through the implementation of EBPs.

Due to the complicated and challenging process of implementing EBPs, many efforts to scale-up effective interventions have fallen short in facilitating systemic and lasting improvements in care [11]. Additional challenges are encountered when implementing EBPs where there is a failure in bridging outer and inner contexts [12]. For example, policies to use EBPs often do not support funding for services. Other challenges arise in low-resource or community settings that serve diverse and vulnerable populations and racial/ethnic minority families. For example, questions remain about how to best adapt EBPs to address different cultures [13] and how to scale-up EBPs in settings with a limited professional mental health workforce [14]. Though the intention of implementing EBPs is to ameliorate mental health disparities, without efforts to focus implementation on vulnerable communities, these disparities might be exacerbated [15]. Given current events, such as global conflicts that are increasing numbers of refugees across the world, and greater racial/ethnic and socioeconomic diversity of families needing mental health services, it is critical to broaden our perspectives to meet the mental health needs of underserved and vulnerable children and families.

Recently, the field of implementation science has emerged to promote and accelerate the systematic uptake of EBPs into community service settings to optimize public health impact [16]. Implementation science is a rapidly growing field as evidenced by multiple investigations of large-scale implementation efforts happening in the United States [17], Europe [18], Africa [19, 20], and Latin America [21]. Several implementation models and frameworks have been developed to describe, guide, and evaluate implementation efforts [22, 23]. We chose to use the Exploration, Preparation, Implementation, Sustainment (EPIS) framework to organize the content and discussions of this colloquium [12, 24]. The EPIS framework is especially helpful to study and guide implementation across different international settings because it is both multilevel (considering outer policy context at the country or local level, and organizational service delivery context such as community based non-profit organizations) and addresses phases and processes to maximize the potential uptake, implementation, and sustainment of implementation efforts for specific populations and settings. A principal objective of the EPIS framework is to guide examination and promotion of the “fit” between the EBP and the implementation service context(s), that is a function of the outer context (i.e., system-level), inner context (i.e., organizational, provider, child/family) and bridging (i.e., bi-directional influences between outer and inner contexts), factors during the implementation process, as well innovation factors that characterize the nature of EBPs being implemented. The EPIS framework has been adapted and adopted in large scale implementation projects in developed countries (e.g., Sweden, Norway) and low- and middle-income countries (e.g., Sierra Leone, Nigeria) and can be easily adapted for other US and European settings [12].

We used the structure and domains of the EPIS Framework to organize the specific content that was presented and discussed during each day of the colloquium. The research projects that were featured provided examples across the continuum of the EPIS, with methodological approaches connected to the Exploration, Preparation, Implementation, and Sustainment phases.

Capitalizing on this unprecedented opportunity to convene a small group of international and multidisciplinary researchers conducting health services, policy and implementation research, our colloquium had the following objectives:

1. Identify common and unique approaches to improve mental health services for diverse and underserved population.
2. Foster and strengthen lasting collaboration between US and French, and pan-European scholars to advance research and practice in children’s mental health services and implementation science.
3. Develop innovative products related to advancing implementation science to address mental health service disparities for children and families.

## Methods

### Colloquium overview

The colloquium, “*Leveraging Implementation Science to Reduce Inequities in Children’s Mental Health Care,”* sought to address how implementation science can improve care for underserved children and families across global contexts and accelerate efforts to increase access to evidence-based mental health treatment. This colloquium was funded by a grant provided by the Borchard Foundation Center on International Education, a private non-profit organization that aims to promote and advance research, education and practice to improve the human condition through international collaboration and scholarship. Participants gathered for a three-day meeting in Missillac, France. The format of the colloquium included a balance of didactic research presentations from each participant along with interactive group learning and strategic product development activities. Specifically, each participant presented on their efforts within their home countries or their global work abroad to reduce mental health care disparities for vulnerable children and families. The EPIS Framework [24] guided the colloquium, after a brief overview of EPIS and its application the first day of the colloquium focused on work the researchers have done regarding the “Outer Context” of healthcare delivery research examples in the U.S. and Europe related to health policy and service system influences on the implementation of evidence-based practices for children and families. The focus of the second day was dedicated to “Inner Context” factors that impact implementation, including implementation in non-governmental and community-based organizations, cultural adaptations and understanding family processes. The final day focused on implementation strategies that bridge the outer and inner contexts, and include additional discussion time for the participants to plan for future collaborations.

### Participants

A total of 11 researchers participated in this colloquium, with 5 (45%) from academic institutions in California, USA and 6 (55%) from academic institutions in Europe. European researchers came from academic institutions in France (*n* = 3), Norway (*n* = 2) and England (*n* = 1). Participants represented a range of academic disciplines and fields including Psychology, Psychiatry, Public Health, Implementation Science, and Health Economics. The participants ranged in their current level of training and education from doctoral students or medical residents (*n* = 2) to early career investigators (*n* = 4) to senior investigators (*n* = 5). In addition, participants were conducting work that examined outer and inner context influences in multiple service settings that play a role in access or delivery of mental health care for children and families, including community-based mental health, child welfare, primary medical care, developmental disability and autism services and school settings.

### Evaluation & Collaboration Measures

An online survey was developed by the colloquium directors (NAS & MLB) and emailed to the colloquium participants. The survey asked participants rate the extent to which they agreed with the following statements: 1) Meeting with researchers from different countries was valuable to my work. 2) Meetings researchers with expertise in disciplines other than my own was valuable to my work. 3) I learned about a new approach to addressing children’s mental health care equity. 4) I plan to incorporate something that I learned from this colloquium in my work. Participants then responded to three open-ended questions about a new approach that they learned from participating in the colloquium, collaborative product ideas and data that they would be willing to share for collaborative projects. To promote ongoing collaboration, participants also indicated their preferences for scholarly products for collaboration and how to maintain communication.

## Summary of Participant Presentations

### Focus of day 1 - outer context factors

Panelists on the first day of the colloquium focused on the unique and shared experiences of policy and service system influences, including insurance, workforce, and public policy, on the implementation of evidence-based practices for children and families.

Panelist 1**:***Using the Exploration, Preparation, Implementation, Sustainment (EPIS) Framework in Mental Health Implementation Research: Results from a Systematic Review and International Case Examples*

#### Dr. Gregory Aarons, University of California San Diego

Dr. Gregory Aarons presented on implementation science frameworks and their relevance to improving care and reducing disparities in child and adolescent mental health. In particular he identified and described recent work identifying the range and types of implementation models, theories, and frameworks [22] and classifying frameworks according to their functions [23]. In order to provide a more specific example, and to set the stage for the colloquium, Dr. Aarons presented and described the EPIS framework named for key phases that guide and describe activities that may take place during the implementation process. He described EPIS phases, the outer context of service systems, the inner context of organizations including structure and process, the role of interorganizational networks, the fit of practices at system, organization, provider, and client levels, and the nature of service innovations and role of intervention developers [24]. The EPIS framework also describes common and unique factors within and across levels of outer (system) context and inner (organizational) context across phases, factors that bridge outer and inner context, and the nature of the innovation or practice being implemented and the role of innovation/practice developers [12]. He stressed the importance of bridging factors in child and adolescent mental health as many services for disadvantaged youth take place in public sector systems.

Dr. Aarons then gave examples of how EPIS is being applied in three large scale implementation initiatives, one using the Interagency Collaborative Teams, strategy to scale-up an evidence-based child maltreatment intervention in a large California county [25], another very large project involving implementing evidence-based, data driven decision-making in 35 sites linking juvenile justice with community behavioral health to address adolescent substance abuse in multiple states [26], and adapting the Interagency Collaborative Teams model to scale-up the Youth Readiness Intervention adolescent mental health intervention in Sub-Saharan Africa [27]. Dr. Aarons then led discussion focused on ways in which implementation science frameworks could be utilized in formulating and enacting improvements in approaches to child and adolescent mental health across a range of settings, countries, and populations.

Panelist 2: *Lay Health Workers as a Scalable Workforce to Reduce Child Mental Health Disparities in Service Access*

#### Miya Barnett, PhD, University of California, Santa Barbara

Dr. Miya Barnett presented on the mobilization of lay health workers (e.g., members of the communities they serve without formalized mental health training) as a workforce solution to decrease child mental health disparities in low-, middle-, and high-income countries. As way of introduction to this issue, Dr. Barnett presented on the substantial gap between the number of individuals who need mental health services and those who access them, and pointed to the inadequacies of the current mental health workforce across a number of global settings to meet these needs. Dr. Barnett then summarized findings from a recent systematic review that she and her colleagues conducted on lay health worker delivered or supported mental health services [28].

Lay health worker models of care were outlined, which included providing outreach and navigation to services, providing auxiliary engagement services, and task-shifting to be the primary providers of treatment. A conceptual model was presented that demonstrated how these different roles impacted the supply and demand drivers of disparities, with outreach/ navigation intended to increase access to care for communities with limited service awareness and stigma (i.e., increasing demand for care) and task-shifting focused on increasing the supply of services in settings with an inadequate professional workforce [14]. Dr. Barnett highlighted how the majority of research has evaluated task-shifting, with lay health workers as the primary providers of treatment in low- and middle-income countries. Studies in low- and middle-income countries were more likely to test the effectiveness of EBPs than the studies with lay health workers conducted in the United States. In line with the EPIS model and topical focus for the day, Dr. Barnett addressed how task-shifting EBP delivery to lay health workers may face outer context barriers in high-income countries due to regulations surrounding training and service provision. Therefore, navigation and auxiliary support services may be more appropriate to increase access for underserved communities.

As an example of one such effort, Dr. Barnett highlighted a current trial she is conducting in the United States to increase engagement in an evidence-based parenting program, Parent-Child Interaction Therapy, for Latino families [29]. In this trial, lay health workers provide engagement services to families to promote improved initiation, adherence, and retention in care, with the intention to improve client and implementation outcomes (e.g., reach of EBP, agency efficiencies). Discussion after the presentation focused on identifying the most appropriate roles for lay health workers in high-income countries in the delivery of EBPs and the implementation supports that are necessary for these roles.

Panelist 3: *Use of Health Policy Initiatives to Address Children’s Health Inequities*

#### Paul Dourgnon, PhD, m.Sc., Institut de Recherche et documentation en Economie de la Santé

Dr. Paul Dourgnon addressed health policies initiatives to tackle children’s mental health inequalities in France. To orient the colloquium attendees to the outer context in France, he first described how mental health care delivery is organized within the French health care system. He then described France’s situation regarding social inequalities in health, mental health and access to mental health care, and recent reforms and programs.

As in most European countries, France implemented a socialized health system after World War II, in the form of a Social Health Insurance, which aimed at providing, and since 2000 has provided universal health coverage to France’s residents. Even undocumented migrants are entitled to the majority of benefits from public health insurance. In France, public health insurance covers three quarters of total health expenditures. The remaining expenditures are covered by private complementary insurance and household out-of-pocket expenses. For care under public health insurance, fee schedules are publicly regulated, but many professionals, mostly specialists, are allowed charge higher rates. These higher rates, along with other billing practices (e.g., requiring upfront payment) have been showed to be strong determinants for social inequalities in access to healthcare services.

France demonstrates therefore a paradoxical picture, ranking very well in terms of population health (as a mean) and low out-of-pocket expenses, but with inequalities in access to specialists, dental care, and mental health services. Furthermore, the French health care system is hampered by poor coordination between healthcare services, complexity and poor utilization of welfare programs. It is also important to note that the very centralized French political system does not favor exchanges between research, especially policy evaluation, and policymaking. Therefore, the French context is characterized by poor knowledge transfer and no real evidence-based policy.

In France, mental health represents a major issue in terms of public health, health care organization and financing, representing the highest expenditure amongst health conditions. The number of patients treated has doubled during the last 25 years, with approximately 15% of children and youth requiring mental health treatment or follow up. Like in other dimensions of healthcare policy, a complex multilevel administrative framework prevails in psychiatric care organization and policymaking. Furthermore, there are regional disparities in the available of services, including a decline in the number of psychiatrists forecast for the future.

Dr. Dourgnon provided a research example of a public audit that was completed on psychiatric care in France in 2017. The main conclusions of the audit highlighted strong disparities in access to treatment, as well as a historical public under-investment in child psychiatry. These challenges with access were especially robust for children, with 2 to 6 times longer waits for children [30]. The report recommended a large investment in child psychiatry. Dr. Dourgnon ended the presentation with a discussion of how to improve knowledge exchange between researchers and policy makers in France and other countries to improve equity in access to mental health services for children.

Panelist 4: *Public Policy Initiatives to Reduce Screen Time in Disadvantaged Youth*

#### Constance Prieur, MS, MPH, Paris Descartes University School of Medicine

Ms. Constance Prieur presented how media exposure in early childhood has consequences on adolescent and adult life. She showed that paucity in available evidence-based public health and prevention interventions in addressing this concern. First, Ms. Prieur presented on research that highlighted the negative impacts of screen and media exposure on infants, toddlers, and their parents. Examples were provided about how media exposure, including use of television and mobile devices impacts parent-child interaction quality, and subsequent socio-emotional and language development [31–33]. Even with these concerns, screen time, including giving mobile devices to toddlers, is extremely prevalent and detrimental to development [34, 35].

Next Ms. Prieur discussed public health interventions surrounding screen time, including publishing recommendations by medical academies and providing written information to parents during pediatric visits to limit all screen time in young children (American Pediatrics Association, académie de médecine). Ms. Prieur explained that some interventions in classroom or at home were taken to reduce time screen, and that a meta-analysis concluded that intervention led to a small but significant effect of reduction of screen time [36]. However, publishing recommendations is generally ineffective. For example, in Canada less than half of families followed the institutional guidelines on media exposure for young children [37]. Discussion surrounded the importance of studying the implementation of evidence-based interventions in promotion of good habits related to media use during childhood, as well as the policy implications for disseminating public health guidelines around screen time.

### Focus of day 2 - inner context factors

Panelists on the second day of the colloquium focused on how practice, client, and organizational factors influence the implementation of EBPs. Focus was placed on how EBPs may need to be adapted to meet the needs of culturally diverse families, to address family processes, and to fit within the EBP implementation context.

Panelist 5: *Adaptations to Evidence-Based Mental Health Interventions for Culturally Diverse Families*

#### Anna Lau, PhD, University of California, Los Angeles

Dr. Anna Lau discussed approaches to the adaptation of evidence-based practices (EBPs) for ethnic minority and immigrant families in the United States, with attention to similarities and differences in strategies reported by intervention researchers and community therapists. EBP adaptations are situated within the EPIS Framework as an innovation factor that can increase the fit of the EBP for a local context with key agents potentially including EBP developers, system or organizational leaders, or front-line community providers. Dr. Lau discussed low fit of EBPs for diverse youth and resultant provider fidelity drift as a potential explanatory process in voltage drop – the diminished effect size of EBPs as they are moved into routine care settings.

Dr. Lau described a conceptual model of indications for cultural adaptations to EBPs [38] and an example of translational intervention research on cultural adaptations to parent training interventions for immigrant Chinese American families [39, 40]. Meta-analytic findings suggest that researcher developed adaptations to EBPs are effective and generate moderate incremental effects over standard non-adapted EBPs [41]. Implementation scientists agree that local adaptations to EBPs are warranted and inevitable [42], but also warn that just as EBPs are subject to the implementation cliff, so too are researcher developed adaptations [13].

Next, Dr. Lau reviewed emerging findings on community therapists’ adaptations to EBPs from the 4KEEPS Study, an observational study examining the sustainment of multiple EBPs in a system-driven implementation in children’s mental health services in Los Angeles County [17]. Data from therapist surveys revealed that the most frequent types of spontaneous adaptations by therapists could be categorized similarly to researcher-driven approaches as Augmenting adaptations involving (1) modifying the presentation of intervention strategies, (2) integrating relevant supplemental content, and (3) lengthening treatment by slowing down the pace. Therapists less frequently endorsed Reducing adaptations that involve (1) omitting intervention components, (2) reordering intervention components, or (3) shortening treatment by quickening the pace [43].

Dr. Lau also reviewed data on reported reasons for adaptations and implications of adaptations for the robustness of EBP delivery. Community therapists report making adaptations for reasons related to clinical issues, developmental level, literacy, and cultural fit. When adapting for cultural reasons it was virtually always Augmenting – to frame EBPs in familiar terms and to intensify teaching new skills by extending pacing [44]. Finally, analysis of session-level therapist descriptions of adaptations and observer rated extensiveness of EBP delivery reveal that modifying presentation adaptations were related to more extensive delivery of EBP strategies, but Extending adaptations were related to less extensive strategy delivery [45, 46]. Discussion after the presentation focused on the value of documenting practice-based adaptations to EBPs (i.e., the ‘Adaptome’ [42]) and translating these findings to inform optimal EBP design, training, and implementation strategies.

Panelist 6: *The Role of Parent and Family Functioning Processes in Youth Mental Health Service Access and Delivery: A Focus on Family Estrangement*

#### Lucy Blake, PhD, Edge Hill University

Dr. Lucy Blake presented research on family estrangement, a term increasingly used to refer to relationships between family members that are characterized by distance in terms of contact, communication and relationship quality. Dr. Blake presented the findings of a literature review of estrangement between parents and their adult children [47], as well as the findings from a survey of 807 individuals who identified as being estranged from a parent, sibling or adult child [48].

Estimated by some researchers to be as common as divorce, the factors that contribute to family estrangement have been identified as being diverse, including: sexual, physical, and/or psychological abuse and/or neglect in childhood; feelings of betrayal; disagreements about romantic relationships and politics; and issues relating to money, inheritance, or business. Family estrangement might also be initiated or exacerbated by physical and/or mental health problems in the family. Given the prevalence, emotional impact and stigma surrounding family estrangement, there is a critical need for mental health counsellors to know how to respond to this issue.

Dr. Blake summarized findings from a qualitative analysis of individuals experiences of receiving counselling for family estrangement [49]. Those who worked with counsellors who were supportive of respondents’ decisions and feelings and did not feel pushed to think, feel or act in a certain way (e.g., to forgive family members, or to initiate estrangement) were reported to be the most helpful encounters. Counsellors who had expertise about estrangement, as opposed to those who reinforced commonly-held assumptions or myths about family relationships (e.g., that mothers are always loving, or that an active and close relationship with a family member is always desirable), were likewise highly valued.

Discussion after the presentation focused on how to move this relatively new field of research forward effectively and efficiently, with the establishment of a definition of estrangement that can be utilized by quantitative, mixed methods and qualitative researchers, as well as those experiencing family estrangement, being paramount. Other avenues of suggested research included the establishment of the mental health needs of individuals experiencing family estrangement, as well as the identification of the training needs of those who work with individuals experiencing family estrangement.

Panelist 7: *Fitting the Context: National Implementation of Trauma-Focused Cognitive Behavioral Therapy (TFCBT) in Norwegian Child Mental Health Clinics*

#### Ane-Marthe Solheim Skar, the Norwegian Centre for Violence and Traumatic Stress Studies

Dr. Ane-Marthe Solheim Skar presented on national implementation efforts to increase trauma screening and evidence-based treatment for post-traumatic stress symptoms in Norwegian specialized Child and Adolescent Mental Health Services (CAMHS). First she identified how trauma has been under recognized within clinical samples in Norway, although systematic screening shows that 79% of children report exposure to potential traumatizing events [50]. Then she described how in 2011, the Norwegian Centre for Violence and Traumatic Stress Studies was commissioned by the Ministry of Health to implement Trauma Focused-Cognitive Behavioral Therapy (TF-CBT) on a national level. TF-CBT is an evidence-based treatment for trauma exposed children with posttraumatic stress symptoms [51] that has been shown to be more effective than therapy as usual in a Norwegian context [52]. Goals of the ongoing implementation project included increasing clinics 1) routine screening for trauma; 2) provider fidelity to TF-CBT, and 3) sustainability of the practices over time.

Dr. Skar then described the phases of the national TF-CBT implementation using the EPIS framework. By 2015, approximately 50% of Norwegian CAMHS took part in the implementation, however without the capacity to scale-up [53]. Several implementation challenges were identified related to the costs of therapist training and supervision, therapist turnover, challenges in obtaining therapist fidelity to and sustainment of the model and a lack of organizational and leadership support.

These challenges have been addressed through two implementation strategies. Firstly, as leadership is important for both implementation, sustainment and turnover intention, the Leadership and Organizational Change for Implementation (LOCI) [54] was selected and adapted to a Norwegian context [55]. Secondly, a less costly TF-CBT training model was implemented. By 2019, approximately 75% of Norwegian CAMHS had implemented TF-CBT.

Finally, Dr. Skar described several future directions of this implementation project. This included an ongoing hybrid III stepped-wedge trial to test the effect of LOCI as an implementation strategy for treatment of post-traumatic stress symptoms in child and adult mental health clinics [55]. In addition, she explained that a future focus is to scale-up the implementation of TF-CBT to the remaining CAMHS, as well as to scale-out to child advocacy centres, which represent a new delivery system and provider population.

The discussion following the presentation focused on several topics. For example, the interaction of outer and inner context for successful implementation, how implementation of TF-CBT applies to reducing inequities in children’s mental health care by providing all children referred to CAMHS with equal opportunities to receive evidence-based treatment for post-traumatic stress symptoms, and challenges related to identifying and retaining children and families in care.

Panelist 8: *Intergenerational Transmission of Health, Long-Lasting Impact of Childhood Conditions on Health and Equity: The Point Of View of an Economist*

#### Florence Jusot, PhD, Université Paris-dauphine (Leda-Legos) & Institut de Recherche et documentation en Economie de la Santé

Dr. Florence Jusot’s presentation covered three primary topics. The first was a discussion about defining health equity through the lens of the Inequality of Opportunities in Health Framework. This framework [56] posits that health inequalities result from a combined contribution of efforts (e.g., lifestyle choices) and circumstances (e.g., biological or social factors that are not chosen by an individual). Inequalities that are primarily driven by circumstances are recognized as “inequalities of opportunity” and should be the focus of intervention efforts. The second topic was about the mechanisms that underlie health inequities. Dr. Jusot shared that examples of health inequities abound in Europe and the U.S. including intergenerational transmission of health behaviors (e.g., tobacco use), health outcomes (e.g., obesity) and health service access and utilization patterns. Potent mechanisms of these health inequalities have been attributed to socioeconomic and familial characteristics [57], thus to circumstances that are beyond individual sphere of responsibility. The final topic was a discussion about effective and efficient strategies to reduce health inequalities. To illustrate potential strategies, Dr. Jusot provided examples in the context of broadening health coverage for low-income families [58] and targeted tobacco reduction initiatives during pregnancy [59]. Discussion that followed this presentation focused on operationalization of “effort” versus “circumstance” as it relates to children’s mental health service access and construction of policies, particularly fiscally-driven, to address mental health inequalities.

### Focus of day 3 – bridging and innovation factors

Panelists on the final day of the colloquium focused on implementation strategies that bridge the outer and inner contexts and explicitly promote design and evaluation of the “fit” between the evidence-based practice and implementation context.

Panelists 9 & 10: *Building Capacity to Access, Engage and Benefit from Evidence-Based Interventions for Autism*

#### Lauren Brookman-Frazee, PhD & Nicole Stadnick, PhD, MPH, University of California San Diego

Dr. Lauren Brookman-Frazee and Dr. Nicole Stadnick co-presented on their inter-connected programs of research that aim to build cross-service system capacity for children with autism spectrum disorder (ASD) and their families to access, engage in and benefit from evidence-based mental health practices. Children with ASD experience high rates of co-occurring mental health symptoms—referred hereafter as children with ASD+ (e.g., anxiety and attention difficulties in addition to core symptoms of ASD)—and require evaluation and treatment from mental health providers [60]. However, mental health providers who deliver psychosocial treatment in community mental health settings have traditionally not received specialized training to provide evidence-based care for children with ASD+ [61]. In response to this training need and the high prevalence of children with ASD+ who seek mental health services, Dr. Brookman-Frazee partnered with community mental health system leaders, providers and families to develop and test the AIM HI (An Individualized Mental Health Intervention for ASD) clinical intervention and provider training model [62]. During the phases of pilot testing, effectiveness and implementation trials of AIM HI, additional needs emerged from the community mental health stakeholders involved. One such need was the insufficient coordination of service systems to facilitate access to and engagement in mental health services for children with ASD+ [63]. An obvious service system to start with to facilitate access is primary care given its central role in global healthcare for children and the fact that primary care providers engage with families regularly (at least annually). Dr. Stadnick’s complementary work through the ATTAIN (Access to Tailored Autism Integrated Care) study [64] has advanced this community-identified need to increase the detection of children with ASD+ in primary care and enhance referral to specialty mental health services. Discussion that followed this joint presentation focused on differences in funding structures of service systems that provide mental health services in the United States and European countries.

Panelist 11: *Using Co-Creation/Co-Production as An Implementation Strategy to Promote Adoption of Research Evidence in Children’s Mental Health and Welfare Services*

#### Thomas Engell, PhD student, the Regional Centre for Child and Adolescent Mental Health, Eastern and Southern Norway

Mr. Engell presented on the use of co-creation for collaborative development and decision making in implementation science and practice. He outlined co-creation as a process involving utilization of diverse knowledge and perspectives from relevant stakeholders to inform decisions. He framed co-creation as a tool for innovation and design which also encapsulates social and democratic considerations, and thus can be relevant for promoting equity in the development of services for children and families. He gave suggestions about how co-creation can be utilized in the exploration, preparation, implementation, and sustainment of innovations and services.

Mr. Engell then turned to a case example from Norwegian Child Welfare Services (CWS). Academic achievement is one the strongest protective factors against future marginalization known [65], and there are pressing needs for effective academic support to help children in CWSs succeed in school. In Norwegian CWSs, however, only 2–3% of services delivered to children and families are evidence-based [66]. One strategy towards increasing adoption and reach of EBPs could be to make interventions less complex and more implementable within the current infrastructure of services (i.e. more feasible, appropriate, acceptable and usable, [67]). In the Knowledge Translation in Child Welfare project [68], co-creation processes with local stakeholders were combined with common elements methodology (i.e., identifying practice-, process-, and implementation elements that are frequently shared by EBPs, [67]) to inform the development of Enhanced Academic Support, a lean and flexible academic support intervention tailored to be implementable in CWSs. The intervention was comprised of four flexible core academic elements, with predefined adaptation alternatives designed for tailoring to contextual and individual needs. Implementation strategies were co-created by the same stakeholders. Preliminary mixed methods result suggest that the intervention is implementable in general CWS practice, and that practitioners use flexibility within acceptable fidelity. Implementation and effectiveness is currently being evaluated [68]. Key learnings were discussed, such as the importance of clarity about what can and cannot be co-created, letting user-representatives (e.g., parents and adolescents) tailor how they contribute in co-creation, and the apparent value of embracing and designing for intervention flexibility as opposed to striving to prevent adaptations.

## Results

Results from the post-colloquium evaluation survey indicated that all colloquium participants (*n* = 11) *strongly agreed* that meeting and spending time and interacting with researchers from different countries and with expertise in disciplines distinct from their own was valuable to their work. Participants *strongly agreed* or *agreed* with the statements that they learned about a new approach to address children’s mental health care equity and that they plan to incorporate a new learning from the colloquium into their work. Examples of new learnings that participants reported that they would apply to their work included co-creation implementation approaches, broader conceptualization of outcomes in health research and use of implementation science frameworks to identify and address needs of specific populations. Regarding preferences for collaborative products, participants indicated that they would like to collaborate on conference presentations (*n* = 10, 91%), manuscripts (n = 10, 91%), grants (*n* = 9, 82%), mentoring activities (n = 10, 91%), and colloquia (*n* = 8, 73%). To facilitate development and execution of these collaborative products, participants preferred the following communication methods: 1) cloud-based sharing and document storage system (n = 10, 91%), 2) email (n = 10, 91%) and 3) interactive, online project management program (n = 8, 73%).

### Common Topics & Observations

The following topics were observed that guided development of our research agenda for prioritizing strategies to address mental health inequities for children.

Topic 1: Barriers to access of mental health care, including access to evidence-based approaches, were common across countries and represented a complex and unique interplay of outer and inner context factors. The primary cross-country barriers to care related to funding structures for mental health services and workforce capacity. Related to funding, presenters identified how challenges in accessing high quality, evidence-based mental health care exist universally, even in settings with policies that allow for universal coverage of services. For example, England has mental health parity and has widely implemented EBPs, such as cognitive behavioral therapy, but long waitlists still limit access to services [69, 70]. Even in Norway, despite impressive and widespread EBP implementation initiatives, challenges were described by presenters related to scaling (up and out) and sustaining EBP implementation. In the U.S., there are significant differences in funding based on the healthcare system that is providing care. For example, medical care to address physical health is typically funded by a combination of private and public health sources whereas mental health care is typically funded by either private or public sources. This can result in more limitations placed on mental health coverage compared to broader health care coverage. Paradoxically, there can be better access to EBPs for children funded by public insurance plans because of increasing policy drives focused on EBP implementation in publicly-funded services [71].

Beyond funding services, participants across countries identified challenges with having an adequate workforce to provide mental health treatment, especially for immigrant populations. Task-sharing with lay health workers, who come from the communities being served, was identified as one potential solution, with the recognition that further research is needed to identify the appropriate roles and implementation supports needed for this workforce [14]. As all of the participants came from high-income countries, there was a recognition that lay health workers should serve in a complimentary role to professional mental health providers with specialized training. Potential roles to address disparities included: 1) providing stepped-care, where lay health workers provided prevention level services and specialists serve more intensive cases; 2) navigation services, especially for individuals with complex health and mental health needs, such as individuals with autism spectrum disorder; and 3) auxiliary services that focus on improving engagement and adherence in treatment.

Topic 2: Another important topic discussed during the conference related to the role of adaptation of EBPs within and across settings. Adaptation has been recognized as inevitable and often necessary to improve the fit of an EBP within the implementation context [42]. Colloquium participants discussed several approaches to adaptations, including researcher driven adaptations to EBP protocols to improve cultural appropriateness for ethnic minority families [38], studying clinician driven adaptations within implementation efforts [43, 44], and co-creation, in which local stakeholders help develop and adapt interventions to be less complex and more implementable [68]. Colloquium participants expressed high levels of interest in co-creation as an example of an implementation strategy that promotes multi-stakeholder collaboration and partnership, which are at the heart of the EPIS framework through the bridging factors.

Topic 3: The final topic that emerged is identifying new opportunities to integrate implementation science approaches. Colloquium participants identified several public health issues related to children’s mental health services that could benefit from applying implementation science frameworks and methods. For example, youth screen time use (C. Prieur) and family estrangement (L. Blake) were discussed as emerging areas that may require new or adapted interventions to address the mental health sequelae that may result. Implementation science is poised to offer systematic guidance through several mechanisms. Examples include: defining the public health problem to be addressed; selecting the EBP or adapting existing EBPs to address the public health problem; identifying or adapting implementation strategies to facilitate EBP uptake; establishing community-academic partnerships to maximize “fit” between EBPs, implementation strategies and implementation context; designing policy and evaluation methods that are nimble to support the implementation and sustainment of efforts. Several colloquium participants were newly introduced to the field of implementation science but reflected on the value and utility of implementation science approaches to their work in children’s mental health services, regardless of the country in which implementation would occur.

## Conclusions

Across the globe, there is an urgent need to improve the access and quality of mental health services for children to promote healthy families and communities. This conference provided an unparalleled opportunity for synergistic work across countries to tackle challenges in implementing evidence-based mental health treatments for the most vulnerable children. The structure and process of this retreat and the survey findings are consistent with recent work that provides recommendations for fostering international collaboration in implementation science and research [72]. A future direction, among the colloquium participants and for the broader research and practice communities, is to identify cross -country and discipline collaborative opportunities to integrate implementation science methods as a vehicle of accelerating the transport of research or practice-based evidence into routine care service contexts.

## Data Availability

Not Applicable.
